# Enhancing radiomics and Deep Learning systems through the standardization of medical imaging workflows

**DOI:** 10.1038/s41597-023-02641-x

**Published:** 2023-10-21

**Authors:** Miriam Cobo, Pablo Menéndez Fernández-Miranda, Gorka Bastarrika, Lara Lloret Iglesias

**Affiliations:** 1 Advanced Computing and e-Science Group, Institute of Physics of Cantabria (IFCA), CSIC - UC, Santander, Spain; 2 Clínica Universidad de Navarra, Department of Radiology, Pamplona, Spain

**Keywords:** Mathematics and computing, Biomarkers

## Abstract

Recent advances in computer-aided diagnosis, treatment response and prognosis in radiomics and deep learning challenge radiology with requirements for world-wide methodological standards for labeling, preprocessing and image acquisition protocols. The adoption of these standards in the clinical workflows is a necessary step towards generalization and interoperability of radiomics and artificial intelligence algorithms in medical imaging.

## Introduction

According to the American Cancer Society it is estimated that around 2 million new cancer cases will be diagnosed in 2023 in the United States^1^. Medical imaging in oncology is the reference to evaluate most cancers, in particular for lesion detection and staging, which proves the need for general standards and guidelines in radiology to advance research in digital diagnosis. Medical images in radiomics play a key role not only in diagnosis, but also in monitoring the progression and development of tumors, in addition to supervising the response to therapy and risk of relapse^2,3^. Throughout the present text, the term radiomics will be used to encompass both classic radiomics and advanced data analysis techniques based on Artificial Intelligence (AI), such as deep radiomics^4,5^.

Recent advances in medical imaging have shown the prospects of quantitative image descriptors to emerge as noninvasive prognosis phenotypes and predictive biomarkers^6,7^. Medical imaging and virtual biopsy are noninvasive techniques in oncology that reach the whole tumor volume^8^, in contrast with genomics and proteomics, which rely on biopsies or invasive surgeries to analyze only a limited sample of tumor tissue that may not be representative of the whole lesion due to its heterogeneity^6^. Radiomics and radiogenomics have shown potential to solve these inconveniences^7,9,10^, but there are several challenges that must be implemented in the workflows of clinical practice. Interestingly, radiomics is not exclusive to oncology, and can be applied to a wide range of medical imaging modalities, from magnetic resonance imaging (MRI), computed tomography (CT), ultrasound, positron-emission-tomography (PET) and single-photon emission computerized tomography (SPECT)^5,11,12^.

The promise of radiomics lies in its potential for noninvasive automated evaluation of medical images. The price will be standardizing the different workflows in image acquisition, preprocessing, annotation, anonymization, metadata, and storage processes. Here we present an overview of current methods in preprocessing and harmonization alongside the limitations of radiomics. Furthermore, we propose guidelines to facilitate standardization and outline future prospects in the field of medical imaging.

## Medical imaging beyond the hospital’s four walls: limitations

The translation of computer vision advances into clinical practice is currently being delayed due to the lack of standardization and harmonization of radiology clinical protocols and workflows^13^, a well-known problem^14^ that calls for a unified approach with the engagement of the industrial sector in the field of radiomics. The potential of P5 medicine (predictive, preventive, personalized, participatory, psycho-cognitive)^15^ to revolutionize the state of the art in medical imaging requires a paradigm shift from individual to collective standards, particularly in data collection and preprocessing. This shift will also enable the transition of research from retrospective studies to clinical applications.

Several reviews of publications discussed by^16^ reveal that most current machine learning models are far from being ready for real-world clinical deployment. These models lack sufficient reproducibility, rigorous validation, generalizability to external datasets, and robustness to translate to clinical practice.

**Fig. 1:**
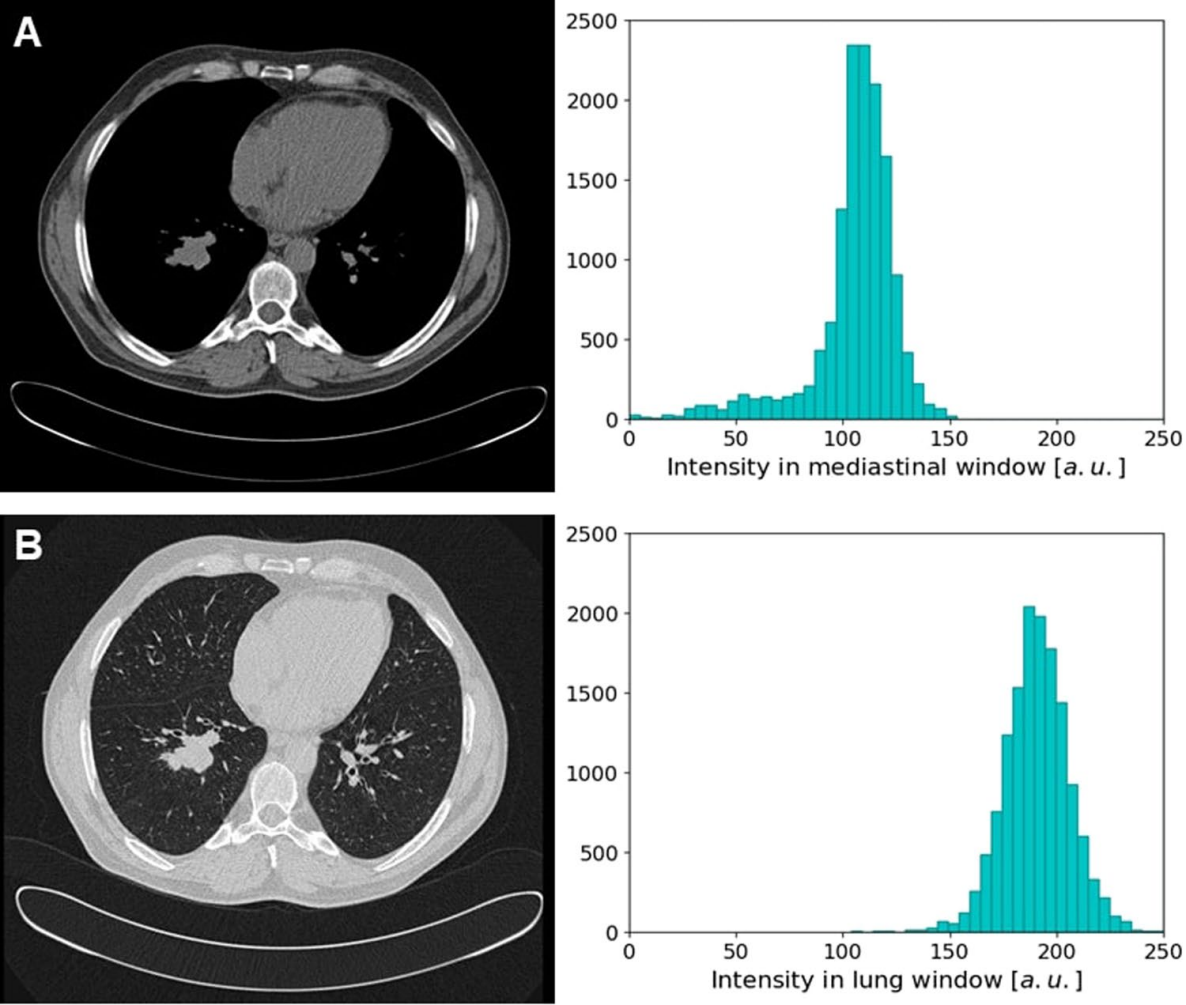
Effect of different preprocessing steps on the same nodule and the corresponding histograms calculated for the nodule mask: (**A**) mediastinal window, (**B**) lung window (a.u. refers to arbitrary units).

There is a wide variability between manufacturers that implement distinct reconstruction algorithms, and institutions that utilize different reconstruction parameters, which may also be customized for each patient^17^. The implementation of standard scanning protocols across institutions will satisfy the urgent need for consistency in the acquisition parameters. Orhlac *et al*.^18^ showed in CT that scanner parameters such as reconstruction kernel or slide thickness influence radiomics texture features. Moreover, Son *et al*.^19^ showed that similar CT protocols and same slice gaps in data from different hospitals lead to an improved performance of machine learning algorithms. Rizzo *et al*.^17^ proposed identifying and excluding radiomic features highly influenced by the acquisition and reconstruction parameters, however this solution may limit the power of radiomics analyses. Image quality is another factor that impacts the performance of radiomics systems, particularly if the equipment has become obsolete compared to modern devices^16^. In case the images come from different sources (manufacturers, hospitals) a similar distribution of “positive” and “negative” cases needs to be ensured to train an AI algorithm^16^. Moreover, preprocessing steps like filtering, resampling and morphological image processing also have an impact on radiomic features, as depicted in Fig. 1, that remains to be further investigated^20^. Finally, for AI systems, data augmentation should not alter the images in a way that the underlying biological or tissue properties are implausible^16^.

There have been some attempts in the literature to provide guidelines to preprocess medical images. Van Timmeren *et al*.^11^ enumerates some of the necessary steps before radiomic feature extraction, such as interpolation, normalization and discretization. However, the authors highlight that many questions regarding these steps remain open. Aerts *et al*.^6^ performed radiomics analysis from the RAW imaging data (before the images are reconstructed), without any pre-processing or normalization, yet a strong dependence of their radiomic signature on tumor volume was later revealed by^21^.

Recently, the ComBat harmonization technique has been applied in several medical imaging modalities, such as lung cancer CT datasets^22^. ComBat harmonization is a batch-effect correction^23^ that aims to suppress batch effects by standardizing the means (location) and variances (scale) of each feature across batches to reduce the batch effect error^24,25^. This algorithm is based on an empirical Bayes approach, originally developed for genomics data^26^, later applied to reducing radiomics variability in PET^27^, and CT^18^. There are other variations of the algorithm, such as longComBat^24^, developed for longitudinal data. Overall, ComBat is intended to harmonize radiomic features, thereby minimizing the impact of different acquisition protocols on radiomic feature extraction, which is particularly useful for retrospective studies, where it would be impractical -or even impossible- to re-image patients to a controlled imaging protocol^22^. Ligero *et al*.^25^ applied ComBat considering different sources of variance as batches: manufacturer-dependent convolution kernel, slice thickness, and the combination of both. Their results showed that ComBat correction minimized radiomics data variability regardless of differences in CT acquisition protocols^25^. In the study by^22^, ComBat harmonization proved to be effective by harmonizing radiomic features extracted from different imaging protocols, although the authors underline that its effect on imaging-feature based predictive models requires further investigation^22^. In fact, research is underway to analyze the power of ComBat harmonization in multicenter studies in various imaging modalities, for example^28^ studied ComBat harmonization on PET/MRI and PET/CT for radiomics-based tissue classification. Furthermore, ComBat is generalizable to other imaging modalities as it makes no assumptions about the origin of the site effects^23^.

The previous examples illustrate the need for general guidelines for medical image preprocessing in computer vision tasks.

The clinical utility of an algorithm highly relies on the quality of the reference standard used in its training and evaluation^16^. Reference standards based on radiologists’ opinion are subjective, especially if established by a single expert, and should therefore be replaced whenever possible by objective reference standards, such as diagnostic tests and pathologic evaluation of biopsies or excised lesions, patient survival or time-to-progression for shorter-term reference standards^16^.

There are several standardization initiatives and imaging protocols investigating homogenization of image biomarkers and radiomic features, such as the Image Biomarker Standardization Initiative (IBSI)^29^, the Quantitative Imaging Network of the National Institute of Health (QIN)^30^, the Quantitative Imaging Biomarkers Alliance (QIBA)^31^, and the European Imaging Biomarker ALLiance (EIBALL)^32^, among others. Harmonization of the extraction and validation of robust radiomic features is essential to achieve results that are reliable and reproducible^18,33–35^, although it does not address the systematic variations between patient subpopulations^16^. The range of different standardization initiatives shows the need to reach consensus among the radiomics research community on joint standards.

Radiomic signatures are intrinsically data driven, which poses several challenges as the high volume of features is susceptible to overfitting and overinterpretation of the derived models^10^. The development of radiomic signatures is significantly affected by underlying dependencies between radiomic features, redundancies and multicollinearity, as outlined by^33^. Machine learning algorithms can be effective to identify unexpected effects, such as volume-confounding features^34,35^. Lately, the lack of biological meaning of current high-throughput agnostic radiomic analyses has raised concerns. Tomaszewski and Gillies^10^ emphasize the need of supporting radiomics with biological validations to gain insights into the casual relationships of the features with the outcomes.

Most published radiomics studies lack independent validations of their signatures beyond a single external test set^10^, which is insufficient for their deployment in clinical practice. Independent validations of radiomic signatures on different cohorts and multiple institutions are hindered by the lack of standardization in medical imaging, although^36^ have already proposed an approach for distributed radiomics. Therefore, to achieve generalization and robustness of radiomic signatures further efforts are required to homogenize image acquisition and preprocessing^18^, in addition to controlling the effect of potential confounders^35^.

Another aspect that hinders the translation of radiomics and AI tools to clinical practice is the black-box nature of most current deep learning systems. Thus, in Europe, the General Data Protection Regulation establishes that individuals have the right to receive a clear and understandable explanation of how artificial intelligence is being used to make decisions that directly affect them^37^. Explainable AI is essential to gain the trust of physicians and understand the reasons behind a prediction or decision^16^. Besides, interpretability can detect biases and problems such as unbalanced data, and explainable models are more robust against adversarial attacks^38^. Post-hoc explanations like saliency maps are insufficient to provide a full explanation of why and how the features are connected and weighted to identify the target lesion. Provided explanations should align with medical knowledge or be supported by clinical evidence^16^. In this regard^39^, introduced Co-12 properties, a high level decomposition of explanation quality, such as completeness, correctness, and compactness. A promising alternative to black-box AI algorithms are Part-prototype models, explainable by design. In this field, PIP-Net (Patch-based Intuitive Prototypes Network) proposed by^40^ opens up a new field of research for explainable AI in medical imaging.

The shortage of large enough datasets to train and externally validate radiomic signatures in prospective multi-center studies also happens for medical AI devices^41^. Several of the devices approved by FDA for diagnostic use were trained on small datasets from a single center or from only two centers^42^. These algorithms are prone to biases and lack of generalizability outside the site where they were trained.

Public databases provide free validation datasets to the medical imaging community, however, as argued by^16^, the QA process for data in a public database is often overlooked. For example, the well-known LIDC-IDRI dataset^43^ includes the manufacturer in DICOM metadata, but not demographic information such as patient age or gender^44^, which can lead to unexpected biases when developing radiomics and machine learning models.

As outlined by^16^, even if a hospital could use a vendor-trained computer-aided diagnosis (CAD) AI tool with multi-institutional data and approved for clinical use, its performance in the local population could not be the same as in the vendor’s specifications. Hence, the hospital would have to evaluate the performance of the CAD-AI tool on their patients in an adjustment phase, achieving a deeper understanding of the CAD-AI performance in the local setting, while reducing unrealistic expectations and improper use of the CAD-AI tool^16^.

To ensure data availability, accessibility and reusability, radiomic signatures demand stability and reproducibility across different hospitals, scanners and acquisition protocols, that is, the adoption of FAIR principles, as described by^45^, in a manner that preserves patient privacy^13^. Data collection must also conform to the ethical considerations and legal framework of the country in which the data were obtained^16^. Standardization extends to validation and evaluation criteria, providing guidelines and contrasted metrics to reduce bias and overly optimistic results hiding the lack of generalization of certain models subjected to highly restrictive data conditions and insufficient reporting^11^. The Radiomic Ontology project^46^ provides a Python library for FAIR radiomics analysis which aims to facilitate the transfer of research efforts to clinical practice.

Despite the mentioned efforts, it is important to note that a consolidated standard in the field of radiomics is still far from being established.

## Towards standardization: guidelines

**Fig. 2:**
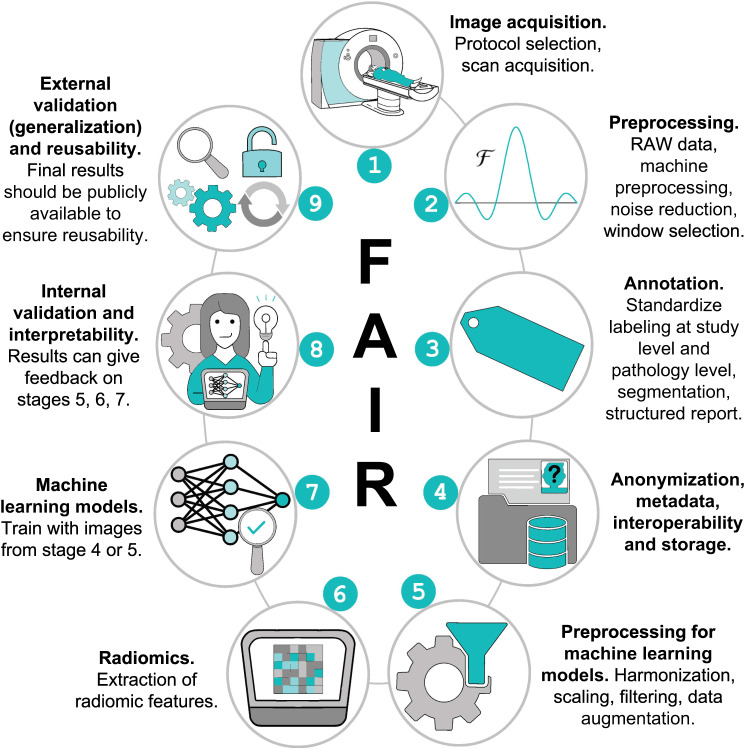
The nine stages of reaching standardization and making medical imaging data as FAIR as possible.

At the moment, there are several public databases available with medical images, such as The Cancer Imaging Archive^47^ or Neurovault^48^. However, the absence of standardization in the format of these databases (i. e. interoperability) hinders simultaneous use of different data sources in the same machine learning algorithm^13^. Thus, the change of paradigm from visual assessment of medical images to computer-aided evaluation demands for methodological standardization of the workflows in medical imaging as proposed in Fig. 2. This standardization should implement the FAIR principles to the extent that the requirements due to the nature of medical images (de-identification, security) allow.

Data collection is a crucial step to create computer vision models and involves different agents within the hospital: radiologists, technicians, nurses, general practitioners, etc. Data interoperability is vital to facilitate research and multicenter studies, therefore all the involved agents in data collection should become aware of methodological standards when these are adopted. We believe radiologists will play a key role in ensuring the correct application of standards and the effective adoption of protocols. There are two levels at which standardization of the workflows in image analysis should be implemented: software (consistency of technical implementation among scanners and manufacturers) and human interaction (coherence between different observers and practitioners)^5^.

At human interaction, we identify two levels at which radiological studies should be labeled: study level (e.g. brain MRI FLAIR sequence, chest radiography AP, etc.) and pathology level (e.g. tumor, benign nodule, etc.). The study level labeling relies on the work of technicians and nurses, who are responsible for the correct categorization of the data according to the type of study modality they have performed. Hence, in the study level labeling, the Series Description parameter in DICOM should correctly include the type of study modality that was carried out. Ultimately, the labeling at study level should be incorporated in the DICOM Study Description and Series Description fields, according to the RadLex lexicon^49^ standard. Therefore, it is essential that this field is homogenized for each DICOM across all hospitals and scanners. In addition, the pathology level labeling should be incorporated into the structured report^50^.

In software, we believe that manufacturers’ involvement in the process of standardization is essential, as they are in charge of bringing the latest technology to the clinic. To ensure their engagement, we propose that all leading radiological societies join forces to request the implementation of the necessary technology from the manufacturers. In particular, we acknowledge that standardization of MRI protocols for MRI-based radiomics is a challenge^51^, due to the inherent versatility of this imaging modality. The experience of^52^ first reported a systematic inventory of MRI technology and personnel. They proposed the creation of a committee of stakeholders (radiologists, MRI physicists, technologists and scientists) committed to establishing and maintaining a standardized imaging strategy, with annual protocol reviews. As for their conclusions^52^, demanded better remote connectivity to MRI systems and automation of exam acquisition, from protocol selection and configuration to parameter modification. In other medical imaging modalities, such as radiography or CT^53–55^, the same process as in MRI could be followed, automating exam acquisition and parameter selection based on the patient’s characteristics.

We propose the following guidelines to ensure generalization of radiomics systems:

- Medical imaging datasets should always incorporate metadata information about the manufacturer and the acquisition protocol.
- Datasets’ anonymization process should retain demographical information (e.g., age, gender, comorbidities, ethnicity) to avoid biases, as long as the patient cohort is sufficient to ensure patient de-identification.
- Datasets that include segmentations should provide metadata describing if the segmentation was manually performed, otherwise information describing the automatic or semiautomatic method that was used should be provided, including values of internal parameters in case of fine-tuning of the algorithm.
- Reference standards should be objective as far as possible, otherwise, independent evaluations should be secured from several experts with an assessment of the inter-reader variability.
- Hospitals should appoint a stakeholder committee within their staff to guide and monitor the standardization strategy, through a QA/QC process.
- All hospitals should adopt the same standards and guidelines to ensure interoperability.
- Radiomics and AI systems should include interpretable explanations in human-understandable terms, similar to medical standards, on how and why they perform predictions or decisions to assist physicians.
- Datasets along with their metadata, and code if exists, should be made publicly available to allow reusability and reproducibility.

Standardization of computational statistics for radiomics-based systems should consider data balancing, sufficient patient population in size and diversity to prevent potential biases, interpretability, biological validation (relation of radiomic signature to cell morphology, density, distribution pattern, etc.^5^), generalization and suitability of performance metrics to the case of use, among other aspects. Ultimately, it is critical to continuously monitor the performance of radiomics systems to ensure their efficiency does not degrade over time, the so-called data drift^56^, as clinical practices, protocols and patient demographics may change, with a corresponding impact on performance.

## Potential and scope of radiomics

The potential of radiomics is currently hindered by the absence of standardization in the medical imaging workflow. There are two factors that hamper standardization. On the one hand, there was no homogeneity in the mathematical definition of radiomic characteristics. This point has already been solved by IBSI, which should be adopted by all institutions. On the other hand, radiological images, despite being based on physical metrics, differ in the capture of the same phenomenon (disease) depending on the machine and the patient. Although this issue cannot be totally solved, it can be alleviated by homogenizing the machines to the same standard, which would be achieved by configuring the same acquisition parameters according to standard protocols, as previously explained. In addition, the establishment of standard protocols would also help to reduce the radiation dose^53,54^. The recommendations that have been proposed here require the engagement of all agents within the hospitals across the world, which may seem unrealistic, given the large number of entities that would have to get involved. Ultimately, we argue that the progressive adoption of these guidelines, under the auspices of radiological societies, will encourage new institutions to adhere to them, and, thus, radiomic signatures will progressively start the transition from research to clinical practice.

Apart from the standardization requirements, the translation of radiomics analyses to clinical practice should be relatively effortless and inexpensive. Firstly, radiomics research is usually based on the studies that are routinely performed to patients and it does not require additional diagnostic techniques. Secondly, radiomics studies do not need expensive or complex equipment since the biomarkers can be easily extracted with the aid of a conventional computer with a Graphics Processing Unit (GPU) and the validation of radiomics signatures can be performed distributedly to preserve patient privacy, following federated learning approaches^57^. To the best of our knowledge, one the first studies that assessed the economic impact of AI as an assistive tool was^58^, who conducted a cost-minimisation analysis in diabetic retinopathy screening to evaluate the potential savings of two deep learning approaches compared to current human assessment, concluding that the semi-automated screening model was the least expensive. For this reason, the field of radiomics in medical imaging has the potential to become a powerful tool in providing universal, high-quality and affordable health care to all, including those in low- and middle-income countries (LMICs) where resources and expertise are limited^59^, with the caveat that biases must be carefully considered in this deployment^60^. On a final note, when standardized protocols are established, technicians will be able to focus more effectively on patient care and image quality^52^.

## Paving the way for future medicine: conclusions

The safe adoption of radiomics and computer-aided diagnosis systems poses as a critical requirement the standardization of protocols and workflows in medical imaging. We have presented guidelines to standardize the workflows in medical imaging, with references to the different levels at which homogenization is required and the hospital personnel involved in each phase. The clinical deployment of radiomics will promote the application of more adapted and personalized treatments to the patient, which will ultimately translate into a more efficient management and distribution of the available resources, likely resulting in cost reductions for health systems. Radiomics based systems have shown potential to analyze patient data and predict future needs, which will allow healthcare providers to plan and allocate resources more efficiently. For this reason, it is necessary to standardize medical imaging workflows as soon as possible, to enable the progressive clinical implementation of radiomics and machine learning tools, and to bring precision medicine to the patient.
